# The Effects of Artificial Intelligence on Implementors’ Fidelity of Instructional Strategies During Handwashing Acquisition in Children with Autism

**DOI:** 10.1007/s10882-023-09937-1

**Published:** 2023-11-15

**Authors:** Brenna Griffen, Elizabeth R. Lorah, Nicolette Caldwell, Donald A. Hantula, John Nosek, Matt Tincani, Shea Lemley

**Affiliations:** 1 Louisiana State University-Shreveport, Shreveport (Louisiana), USA; 2 University of Arkansas, Fayetteville, AR USA; 3 Temple University, Philadelphia, PA USA; 4 Guiding Technologies, Philadelphia, PA USA

**Keywords:** Artificial intelligence, Autism, Handwashing, Fidelity

## Abstract

Handwashing is a vital skill for maintaining health and hygiene. For individuals with intellectual and developmental disabilities (IDD), such as autism spectrum disorder, evidence-based strategies, such as prompting and task analysis, may be effective in teaching these skills. Due to the shortage of experts who teach individuals with IDD skills such as handwashing, staff working with children need a means of ensuring these instructional strategies are implemented with fidelity. This study examined the effects of a tablet-based application that used artificial intelligence (GAINS®) on four behavior technicians’ implementation of least-to-most prompting, total task chaining, and time delay during an acquisition of handwashing program with young children with autism. All four technicians increased fidelity immediately upon using GAINS and all four technicians reached mastery criteria within the shortest number of sessions possible. One child participant met mastery criteria, two showed some gains, and one demonstrated a high degree of variability across sessions. Limitations of the least-to-most prompting procedure, user design, considerations and directions for future research and practice are discussed.

Hand hygiene is one of the most effective ways to prevent illness and keep a person healthy, as hands are one of the most common transmitters of pathogens (van Niekerk et al., 2021). According to the Centers for Disease Control (CDC; 2022), handwashing can prevent one in three diarrhea related illnesses and one in five respiratory illnesses, including the common cold, the flu, and COVID-19. Individuals with an intellectual and developmental disability (IDD), such as autism spectrum disorder, may be especially vulnerable to illness due to poor hygiene practices (Deochand et al., 2019; Schieve et al., 2012). For these individuals, parents and caregivers, such as teachers and behavioral clinicians, can play an important role in teaching children to engage in preventative measures, such as handwashing (Ceglio et al., 2020; CDC, 2022). However, effective instruction for children with IDD designed to teach hygiene skills, such as handwashing, often requires individualized educational programs using evidence-based practices (Deochand et al., 2019).

In order to produce desired outcomes, individualized instructional practices for individuals with IDD must target both skill and motivational factors (Deochand et al., 2019). For this reason, methods of applied behavior analysis (ABA) can be used to teach handwashing to children with IDD (Wertalik & Kubina, 2017). For example, Walmsley et al. (2013) evaluated the use of least-to-most prompting and reinforcement on the acquisition of handwashing in five young adults with a diagnosis of a developmental disability. The results indicated that prompting and reinforcement produced a reliable acquisition of handwashing in all five participants (Walmsley et al., 2013). Although this study establishes some preliminary support for handwashing instruction in adults with IDD, a recent systematic literature review of interventions supporting health related routines identified only four studies using behavioral interventions to increase participation or quality of handwashing in children with IDD (St. Joseph & Machalicek, 2022).

While there is not an abundance of literature on the use of behavioral intervention strategies like prompting, total task chaining, and time delay for hand hygiene specifically, these methods of behavioral intervention are established evidence-based best practices according to the National Clearinghouse on Autism Evidence and Practice (NCAEP) and can be extended to self-help skills, such as handwashing (Steinbrenner et al., 2020). A practice can only be labeled as evidence-based by the NCAEP if it has a sufficient number of empirical demonstrations of efficacy as evidenced by (a) two or more high quality group designs studies conducted by at least two different research groups, (b) five or more high quality single case design studies conducted by at least three different research groups with a total of at least 20 participants across studies, or (c) one or more high quality group design studies and at least three high quality single case design studies conducted by at least two different research groups (Steinbrenner et al., 2020). Least-to-most prompting, which has been used to teach handwashing (Walmsley et al., 2013), involves systematically progressing through a hierarchy of prompts, each providing more assistance, until the target skill is completed correctly (Steinbrenner et al., 2020). The least-to-most prompting procedure contains a minimum of three levels with the initial level, or independent level, requiring no prompts. The intermediate levels contain increasingly intrusive prompts until reaching the last level, or controlling prompt, which ensures consistent and correct responding (Sam & AFIRM Team, 2015a).

Research suggests that prompting can be effective in teaching skills across many outcome areas including communication, social, academic, cognitive and vocational (Steinbrenner et al., 2020). Task analysis and total task chaining are evidence-based practices where a complex task is broken down into multiple individual steps and then taught simultaneously until independence on all steps is reached (Cooper et al., 2020; Sam & AFIRM Team, 2015b). Task analysis has been successfully used in studies to increase participants’ skills in communication, peer interactions, academic performance, and vocational training (Steinbrenner et al., 2020). Time delay is a procedure in which a constant or progressive amount of time is given after a cue and before the controlling prompt is initiated. It is used to systematically fade prompts and reduce prompt dependency (Cooper et al., 2020; Sam & AFIRM Team, 2015c). Studies have shown that time delay can increase participants’ skills in communication, joint attention, play, school readiness, and vocational tasks (Steinbrenner et al., 2020).

However, simply identifying evidence-based practices is not sufficient. For practices to be effective, they must be administered by qualified individuals with fidelity (Steinbrenner et al., 2020). Fidelity of implementation is the extent to which an intervention or instructional program is delivered as intended or designed (King-Sears & Garwood, 2020). Higher treatment fidelity has long been associated with improved student outcomes (King-Sears & Garwood, 2020; Nelen et al., 2021; O’Donnell, 2008). In contrast, omitting steps or modifying evidence-based practices compromises the integrity of interventions, limiting the desired impact on students. In addition, low rates of fidelity can lead clinicians to make erroneous decisions that evidence-based practices do not work and abandon them instead of correcting implementation errors (King-Sears & Garwood, 2020). In even worse cases, the lack of acquisition may be attributed to the individual student’s abilities, putting them at risk for more exclusionary educational practices and lowering their self-esteem (Sanetti & Luh, 2019).

Training individuals to implement evidence-based practices with fidelity can be costly, both in time and in monetary resources (Neely et al., 2016; Wainer & Ingersoll, 2013). Traditionally, fidelity monitoring in behavior analytic services requires allowing staff time to attend regular supervision until a satisfactory level of fidelity and competence is achieved (Sanders et al., 2020). Due to the demanding nature of daily tasks, many therapists report lacking time to dedicate to training programs (Wainer & Ingersoll, 2013). For this reason, clinics often opt for training to occur in a onetime workshop format (Neely et al., 2016). Although this format is cost and time effective, it does not produce lasting behavioral change, which leaves many professionals still unable to consistently deliver interventions with a high degree of fidelity (Robinson, 2011; Schepis et al., 2003). Even credentialed paraprofessionals who have met minimal competency standards may not be sufficiently prepared to deliver behavior analytic programs consistently over time. Whenever programs are implemented with low rates of fidelity, it can lead to staff frustration and burnout (Fixesen et al., 2005). This may contribute to the critical shortage of qualified professionals who can administer behavioral programs to children with developmental disabilities (Yingling et al., 2022). Therefore, it is vital that efficient systems of providing effective and sustainable training are identified or developed (Neely et al., 2016).

Artificial intelligence (AI) systems may provide one such solution and potentially mitigate some of the obstacles to training. While there is little agreement on a definition of AI at an academic, government, and community level, AI systems are generally understood to be a machine that is capable of imitating intelligent human behavior and are most often used in tasks that involve complex human actions, such as learning, analyzing, synthesizing, and adapting (Hopcan et al., 2022). Broadly speaking AI is a field of computer science that deals with intelligent machines that imitate intelligent human behavior (Sadiku et al., 2021). Examples of the most basic AI systems include “smart” technologies such as phones, refrigerators, and cars (Kelly et al., 2023). AI systems are generally divided into three categories. First, Artificial General Intelligence (AGI) is presently theoretical but will be able to transfer learning across a variety of scenarios. Second, Artificial Narrow Intelligence (ANI) which includes technologies that use voice recognition by way of machine learning. ANI systems include Apple Siri or Amazon Alexa and are limited in their ability to transfer knowledge across tasks. ANI systems rely on machine learning (ML) for functionality. Finally, Artificial Super Intelligence (ASI) which is more intelligent than human capabilities and in theory will be able to pioneer discoveries in science and creative fields (Kelly et al., 2023).

Some forms of AI, such as ANI systems, rely on ML and natural language processing (NLP) for functionality. ML includes a broad range of algorithms and statistical models that allow the intelligent agent to find patterns, draw conclusions, and perform tasks without specific instructions or explicit programming. Essentially, ML allows AI to “generalize” information. It responds to in-the-moment changes that are occurring in the environment. For AI to be of use to us it needs to communicate in our *language*. NLP works to translate human language to computer language and vice versa. Voice recognition systems are an example of AI that use NLP to function (Sadiku et al., 2021).

AI tools are used all around us, such as in social media platforms like Instagram, Facebook, and LinkedIn. AI technologies are harnessed in the advertising we see and marketing we consume. Many of us carry AI in our hands every day, using smartphones and smartwatches. AI systems are used in both the medical and criminal justice fields (Sadiku et al., 2021). When applied to instructing students with complex needs, AI may be able to personalize learning, make the learning environment more effective, guide implementors through instructional activities, and increase active student engagement in learning (Drigas & Ioannidou, 2012). Currently, AI is used to teach a variety of skills across many developmental domains, including communication, social skills, safety skills, literacy, academic skills, daily living tasks, and fine motor skills, to children with autism spectrum disorder (Rehman et al., 2021). In addition, AI monitors students’ acquisition of skills in real-time throughout the learning process and provides feedback on student performance (Hopcan et al., 2022). The individualization function of AI may be especially efficacious for individuals with disabilities by increasing student independence while supporting instructors in addressing individualized needs. Although the research on using AI with students with disabilities has increased in recent years, there remains insufficient research on the topic to guide researchers and teachers to make effective decisions in its use (Hopcan et al., 2022). Without guidance in how to use the tools that AI provides effectively, its applications and widespread usage will continue to be limited.

While AI is promising in terms of its ability to alleviate many of the barriers in training high quality ABA instruction, at the time of this writing there are no published evaluations of AI’s ability to provide therapists or teachers with low-cost and instantaneous feedback on the use of evidence-based best practices, such as prompting and reinforcement (Hopcan et al., 2022). While evidence is lacking on the use of AI to provide in-the-moment feedback to implementors of ABA practices, we can glean how AI could be used in this way from how AI may be used in our everyday lives. For example, when using a global positioning system (GPS) to guide our travel, the system gives feedback and directions on an individual's navigational performance, often synthesizing information in the moment. Similarly, the technology presented in this study provides therapists with in-the-moment instructions on how to use evidence-based practices dependent on the learner’s performance. So, for example, if a learner is unresponsive to a prompt level, the system will give the instruction to provide a more effective prompt. If the learner responds to a given instruction correctly, the AI system will provide the therapist with the direction to provide reinforcement for performance. While the instructional systems are initially individualized by a supervisor such as a Board-Certified Behavior Analyst (BCBA), for example the prompting hierarchy used for each respective learner, the use of the technology removes the need for close monitoring in terms of the delivery of evidence-based practices.

One such app that uses AI to provide on the job coaching to therapists is the AI software platform Guidance, Assessment, and Information Systems (GAINS^®^; Guiding Technologies Corp., 2022). GAINS provides personalized learning through automated, detailed, step-by-step guidance, during program implementation, that adapts in the moment to learner responses. It supplies real time coaching, similar to how a clinician would provide supervision and training to a therapist but does not require that a qualified clinician be present, either virtually or in person, during sessions. All of the personalization in prompting and skill selection occur in the “back end” when the learner is not present. GAINS works similarly to “bug-in-ear” technology in that the interventionists wear headsets to receive second-by-second guidance on how to implement a specified instructional program, while simultaneously collecting data on learner performance. GAINS provides guidance by adjusting the next step in the sequence based on learner responding. For example, in a handwashing program, the initial step is “Turn on the faucet.” If the interventionist indicates (+), the next step in the handwashing sequence would be introduced via audio and visual instructions. If the interventionist selects (-), the audio and visual instructions would begin leading the interventionist through the least-to-most prompting hierarchy (see Fig. 1 for example). While GAINS offers a promising new approach to ensuring high fidelity ABA implementation, empirical evaluation of the application's effectiveness is lacking.

**Fig. 1:**
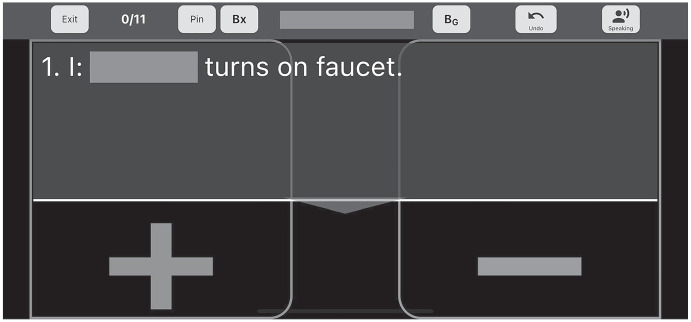
GAINS instructional screen

Given the need for an evaluation of the effectiveness of AI to provide on the job coaching to therapists implementing behaviorally based evidence-based practices, the purpose of this study was to evaluate the use of GAINS on the implementation of handwashing instruction to young children with a diagnosis of autism. As a secondary dependent measure, data were also collected on child participant acquisition to determine if least-to-most prompting, time delay, and total task chaining were effective instructional strategies for handwashing.

## Methods

### Participants

The participants included four children, aged five to six years old, paired in a dyad with their adult therapist. Child participants were selected based on scores of the Assessment of Basic Language and Living Skills- Revised (ABLLS-R; Partington, 2015). Child participants’ ABLLS-R scores and demographic information are displayed in Table 1. The ABLLS-R provides a comprehensive review of 544 skills in 25 skill areas, including language, social interaction, self-help, academic, and motor skills that most typically developing children acquire prior to entering kindergarten. An overall total score of 544 is possible, with a max score of 27 for imitation and seven for grooming. Therefore, the child participants’ scores indicated a lack of independence in all skill areas, including self-help skills, such as handwashing, teeth brushing and hair combing. Each child’s primary therapist was selected as the therapist participant. Therefore, inclusion for therapist participants included a history of working with the child participant and Registered Behavior Technician™ (RBT) certification since this was a requirement at the clinic where the study took place. For the purposes of this study, participants were divided into dyads consisting of one therapist and one child. Dyad One was Antonio and Mary. Dyad Two consisted of Mason and Blake. Ian and Haley were assigned as Dyad Three. Dyad Four was Max and Carlos. Therapist participant demographic information is listed in Table 2.

**Table 1:** Child Participant Information

Name	Age	Sex	Diagnosis	Primary Language	Race/Ethnicity	ABLLS-R Scores
Antonio (Dyad One)	5 years, 11 months	Male	ASD	Spanish	Hispanic	Total – 35 Grooming- 1 Imitation- .5
Mason (Dyad Two)	6 years, 7 months	Male	ASD	English	White	Total- 142 Grooming- 0 Imitation- 17
Ian (Dyad Three)	6 years, 1 month	Male	ASD	English	White	Total- 247 Grooming- 4 Imitation – 18
Max (Dyad Four)	5 years, 2 months	Male	ASD	English	Black	Total- 32 Grooming- 0 Imitation- 4

**Table 2:** Adult Participant Information

Name	Age (in years)	Sex	Race/Ethnicity	Educational Background	Years of Experience with Children
Mary (Dyad One)	24	Female	White	Bachelor’s Degree	1–3 years
Blake (Dyad Two)	20	Female	White	High School	5 + years
Haley (Dyad Three)	25	Female	White	Bachelor’s Degree	1–3 years
Carlos (Dyad Four)	21	Male	Hispanic	Associate Degree	1–3 years

Informed consent was obtained through a face-to-face meeting with each therapist participant and the parent of each child participant. They were informed of the nature of the study, data collection and retention methods, and how the data would be used in the future. Following the meeting, each parent and therapist signed a written consent form.

### Setting and Materials

Sessions took place in the bathroom of the privately owned ABA clinic where each child attended therapy. The materials used during each session included the bathroom sink, an automatic soap dispenser hung on the wall, a mirror above the sink, a paper towel dispenser mounted on the wall, and a trash can placed beside the sink. A small child-size step stool was used for the shorter child participants (Antonio and Mason) to allow them to reach the sink independently. The only people in the room at the time of the session were the two members of the dyad and the primary data collector.

#### GAINS Technology

The intervention involved the use of GAINS, an AI software platform described in the introduction. Since GAINS is a tablet-based software application, the interventionists accessed it through Apple^®^ iPad Minis (2nd Generation). In addition, they wore AfterShokz^®^ OpenMove™ Wireless Bluetooth headsets to access the audio instructions. A Flic 2^®^ Smart Button with Bluetooth capabilities was adhered to one side of the mirror within reach of the therapists but out of reach for the child participants. This Bluetooth button was used because the interventionists' hands would often become wet during the session, making it difficult to tap and swipe on the iPad accurately. One tap of the Bluetooth button indicated a correct (+) response, while two taps indicated an incorrect (-) response. To go back to the previous step, the user would press and hold the Bluetooth button. The GAINS application was started at the beginning of the session and the iPad was placed on a shelf in the bathroom, out of reach of the participants, while handwashing took place.

### Experimental Design

This study used a concurrent multiple baseline across participant design (Ledford & Gast, 2018), consisting of baseline and intervention phases. Dyads were selected to move from baseline to intervention when the therapist fidelity data demonstrated stability across three consecutive sessions. Mastery criteria consisted of the therapist scoring 80% or higher implementation fidelity across three consecutive sessions. When one therapist reached this criterion, they continued in intervention to study the effects of procedures on the child participant, and the next therapist entered the intervention phase. Mastery criteria for the child was set at 100% independent and accurate responding across three consecutive sessions. Data collection continued for the duration of time allotted for the study or until both the therapist and the child met mastery criteria.

### Measurement Systems

#### Dependent Measures

##### Implementor Fidelity

The primary dependent measure was the therapist’s correct use of least-to-most prompting, time delay, data collection and the task analysis during each handwashing session as measured by a percentage of correct responses on the Implementer Fidelity Data Sheet included as Appendix A. The Implementor Fidelity Data Sheet was developed by the first author by identifying the critical components of the intervention and then developing a component checklist to record fidelity for each step of the task analysis (O’Donnell, 2008). The critical components were prompting hierarchy, time delay, data collection, and adherence to the task analysis. An eleven-step task analysis of handwashing was used throughout the study (see Appendix B). It was developed using the handwashing steps included on the CDC (2022) website. For a step to be counted as correct, the therapist had to complete all four components by 1) using the least intrusive but most effective prompt, 2) providing a three-second time delay for the child participant to respond before prompting, 3) recording the correct prompt level used, and 4) proceeding to the next consecutive step in the task analysis. If a therapist pressed the Bluetooth button incorrectly (i.e., one time instead of two times) without backstepping, an incorrect prompt level would be recorded and would be marked on the Implementor Fidelity Data Sheet. The number of correct components was divided by the total number of components and multiplied by 100 to create a percentage of correct implementation for each session. The first author, who was a BCBA and doctoral student, served as the primary observer and watched each handwashing session live while documenting responses on each step of the Implementor Data Sheet using pen and paper. In addition, all sessions were video recorded on a separate tablet device. When necessary, the primary observer replayed the session via video recording after completion to ensure the accuracy of data collection.

##### Child Data

In addition to implementor fidelity, the primary observer also recorded the level of prompting needed for the child to complete each step of the task analysis on the Implementor Fidelity Data sheet (see Appendix A). An independent response was recorded when the child initiated and completed the step without prompting within the three second time delay. If the child did not initiate the step, it was counted as prompted. The total number of independent responses was divided by the total number of responses (eleven) and multiplied by 100 to obtain a percentage of independent responses for the session.

##### Social Validity

Following completion of the study, each therapist completed a System Usability Scale survey (Bangor et al., 2008) designed to assess how using GAINS compared to typical instruction methods in acceptability, ease of use, and therapist preference. The survey consisted of approximately 30 questions using a Likert-type scale with responses being strongly disagree, disagree, neutral, agree, or strongly agree. A copy of the survey is included in Appendix C.

#### Data Analysis

Data collected on both dependent variables (therapist fidelity and child independence) were displayed in graphical format and analyzed with visual analysis (Ledford & Gast, 2018). Descriptions of level, trend, variability, consistency, and immediacy are provided. In addition, the magnitude, or amount of behavioral change, was analyzed by comparing the amount and consistency of change across conditions (Ledford & Gast, 2018). Tau-U was used as a measure of effect size. Tau-U was selected because it can adjust for baseline trends, accommodate smaller data sets, and correlates well with other measures of effect size. A score of 0.20 or less indicates a small change. Scores between 0.20 and 0.60 indicate a moderate change, while scores between 0.60 and 0.80 indicate a large change. Any score above 0.80 represents a very large change (Vannest & Ninci, 2015). The usability survey allowed respondents to choose from the following answer choices: strongly disagree, disagree, neutral, agree, and strongly agree. After completion of the survey, each response was assigned a numerical value ranging from 1 to 5 with 1 being “strongly disagree” and 5 being “strongly agree.” Responses were compiled, and data were analyzed using an average and range of responses for each question.

#### Interobserver Agreement

All sessions were video recorded using a separate tablet with video recording capabilities. A sample of the sessions was selected randomly to be used for interobserver agreement (IOA). A BCBA and a graduate student in ABA not associated with the study watched recordings of the selected sessions and collected IOA data. Both observers were trained in data collection by the primary author. IOA were calculated by taking the number of agreements divided by the number of agreements plus disagreements multiplied by 100 to obtain a percentage of agreement. IOA were collected in at least 33% of sessions in all phases for all participant dyads. For Dyad One, IOA for the primary dependent measure was 84.09% (range 84.09%) for baseline and 97.72% (range 93.18–100%) for intervention. For Dyad Two, IOA for the primary measure was 82.95% (range 77.27- 88.63%) for baseline and 99.24% (range 97.72–100%) for intervention. For Dyad Three, IOA was 89.39% (range 86.36- 93.18%) for baseline and 97.16% (range 93.18–100%) during intervention. IOA for Dyad Four was 85.14% (range 79.55- 88.64%) for baseline and 92.61% (range 86.36- 97.73%) for intervention.

### Procedures

Throughout the course of the study, handwashing was taught using total task chaining, and a three second time delay with a least-to-most prompting hierarchy (Cooper et al., 2020). Social praise from the therapist was used as a consequence for correct responding, which was standard practice at the clinic. These specific instructional methods (total task chaining, time delay, and least-to-most prompting) were selected based on the recommendation of each child participant’s BCBA. Each session consisted of completing the handwashing task analysis one time. Sessions varied in length, but generally lasted between two to five minutes, depending on how much assistance each child participant needed. Sessions were conducted during times in the day that the child would normally be washing their hands (i.e., before and after meals, after completing a toileting routine, after playing with sand, etc.). Therefore, the number of sessions conducted per day varied between one and four. Since handwashing happens frequently throughout the day, it can be assumed that participants washed their hands outside of study sessions across multiple settings. However, it is unknown what, if any, prompting or teaching procedures were used with the participants outside of sessions.

#### Baseline

Baseline was a treatment as usual procedure. Prior to beginning the first session, each therapist was given a copy of the handwashing task analysis (included as Appendix B) on paper and instructed to follow it using total task chaining, least-to-most prompting, and a three second time delay with the child participant. Since all therapists had previous training on prompting methods, no additional feedback or information was provided. This was designed to replicate how a standard handwashing program might be implemented during a typical ABA session in the center. During each session, the therapist took the child to the bathroom and completed the task analysis. They collected data on the prompt level used on each step of the task analysis as they typically would, and no feedback was given on their fidelity or the child’s performance on the steps. Data were collected by the first author, as the independent observer, while observing the session.

#### Intervention

##### Pre-Intervention Training

Prior to beginning intervention, the therapist was given an iPad with the GAINS application. The therapist was given a chance to practice using the technology by putting on the headset and working through a sample program that included a task analysis of toileting procedures with a sample child participant. They were allowed to view the program and work through the sample until they felt comfortable using the application and Bluetooth button. All therapists stated they felt comfortable after going through it one time and spent no more than five minutes using the program. In each case, the first author used gestural prompts to show them how to navigate to the correct program, initiate the first step, and how to backstep if needed. Neither the program nor the sample child participant was related to the study objectives in any way.

##### Intervention Sessions

Prior to beginning handwashing, the therapist started the GAINS application and put on the Bluetooth headset. They placed the iPad on a shelf in the bathroom until completion of the handwashing instruction. The GAINS application was pre-programed with the same handwashing task analysis as baseline and gave the therapists audio instructions to complete the handwashing task analysis using a three second time delay with least-to-most prompting for each step of the task analysis. It began with a set-up phase with the instructions, “At bathroom sink, stand behind [Name]. Say ‘time to wash hands.’ Press next to continue.” The therapist would press the Bluetooth button on the mirror to continue. For each step of the handwashing task analysis, an operational definition for an independent response was given. If the child responded independently, the therapist pressed the Bluetooth button once, an independent response was scored by the GAINS app, and the next step in the task analysis was defined. Therapists provided general social praise (such as “great job!”) if the child responded independently. If the child did not respond independently, the therapist pressed the Bluetooth button twice and the app provided an operational definition for a gestural prompt. If the child emitted the correct response based on the gestural prompt, the therapist pressed the button once and moved to the next step in the task analysis. If the prompt did not evoke a correct response, the therapist pressed the button twice and the GAINS app provided an operational definition for a partial physical prompt. Instruction continued in this manner until the least restrictive, yet most effective prompt was identified. Then the next step in the task analysis was introduced. This process was followed for each step of the handwashing task analysis until all steps were complete. Although it did take approximately one to two seconds for the therapist to listen to the instructions provided by the GAINS app, it did not cause significant delays due to the time delay procedures already in place. For example, while the therapist was listening to the audio instruction “[Name] turns on facet”, the therapist was providing time for the child participant to respond independently. The GAINS program stored data on which prompt level was used at each step. Implementor fidelity data were collected by the first author while observing the session using the same data collection sheet as baseline. Mastery criteria was set at 80% fidelity over three consecutive sessions, which is consistent with other instructional programs for children with disabilities (Nelen et al., 2021; O’Donnell, 2008). When a therapist achieved these criteria, they continued in intervention to study the effects on the child participant’s acquisition of handwashing skills, and the next therapist began the intervention. Intervention sessions continued for the duration of the study (which varied by participant) or until the child achieved 100% independence in handwashing across three consecutive sessions.

## Results

**Fig. 2:**
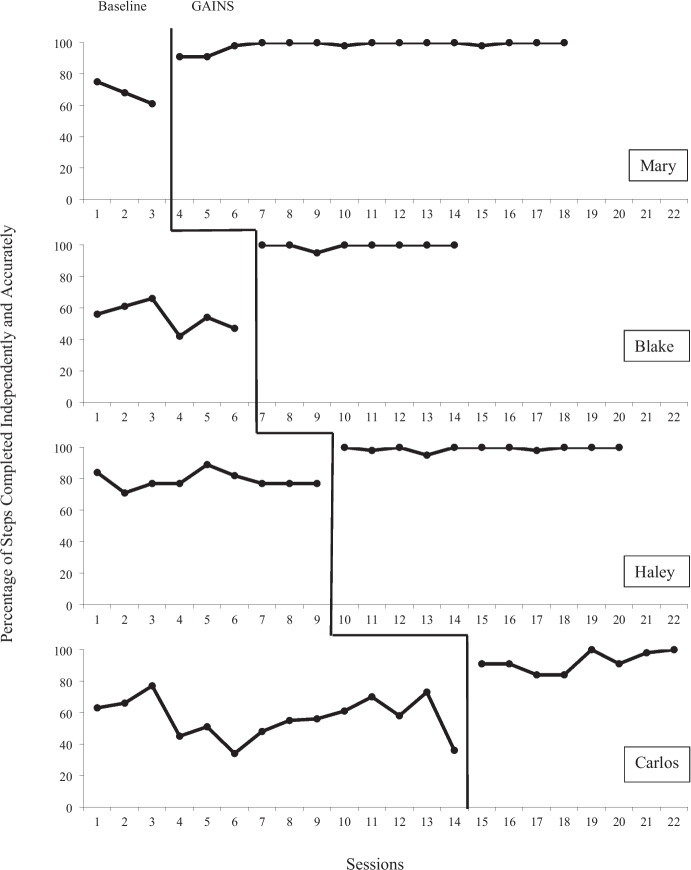
Therapist Percent of Steps Completed

**Fig. 3:**
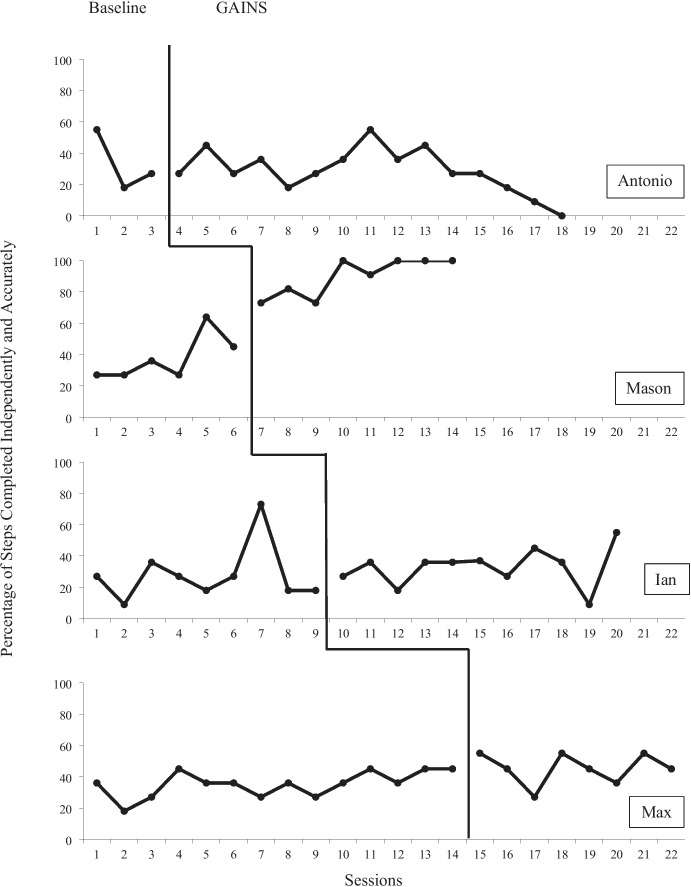
Learner Percent of Steps Completed

Figure 2 shows the data for each therapist’s fidelity of implementation and Fig. 3 represents the data for acquisition of handwashing skills for each child participant. Overall, all four therapists showed an immediate increase in fidelity of implementation during the intervention that was maintained throughout the duration of the study. Two therapists showed a high degree of variability during baseline, which stabilized during intervention. In addition, all four therapists met mastery criteria within three sessions. A weighted Tau-U score for therapist fidelity was calculated to be 1.0, a very large effect size. One child participant mastered all steps of the handwashing task analysis, two showed slight gains, and the other demonstrated variability in performance throughout the study. A weighted Tau-U score for child acquisition of handwashing skills was calculated to be 0.44, a moderate effect size.

### Dyad One (Mary and Antonio)

Mary (the therapist) averaged 68% (range 61–75%) fidelity in baseline with a downward trend. After using GAINS, she averaged 98% (range 91–100%) fidelity during intervention with an upward trend. She required only three sessions to mastery. There is a clear indication of immediacy, and her data show a high magnitude throughout intervention. Tau-U was 1.0, a very large effect size. Antonio (the child participant) averaged 33% (range 18–55%) steps completed independently during baseline and 29% (range 0–55%) during intervention. Antonio’s data illustrate some variability with a downward trend during intervention. Tau-U was –0.07, which indicates that the intervention did not produce a substantial change.

### Dyad Two (Blake and Mason)

Blake (the therapist) averaged 54% (range 43–66%) fidelity in baseline with a downward trend. She averaged 99% (range 95–100%) during intervention. She required three sessions to reach mastery with a clear indication of immediacy and high magnitude throughout intervention. Tau-U was 1.0, which indicates a very large effect size. Mason (the child participant) averaged 38% (range 27–64%) steps completed independently in baseline. He averaged 90% (range 73–100%) during intervention and reached mastery criteria in eight sessions. His data show immediacy, a clear upward trend, and a high magnitude. Tau-U was 0.83, which indicates a very large effect size. Sessions were ended for Mason when he reached mastery criteria for completing the entire handwashing task analysis at 100% independence across three consecutive sessions.

### Dyad Three (Haley and Ian)

Haley (the therapist) averaged 79% (range 71–89%) fidelity in baseline with a fairly stable trend. She averaged 99% (range 95%-100%) during intervention. Her data show indications of immediacy and high magnitude during the intervention phase. She required three sessions to mastery. Tau-U was 1.0, a very large effect size. Ian (the child participant) averaged 28% (range 9–73%) of steps completed independently during baseline and 33% (range 9–55%) during intervention. His data show a high degree of variability throughout with no clear trend, while Tau-U of 0.36 indicates a moderate effect size.

### Dyad Four (Carlos and Max)

Carlos (the therapist) averaged 57% (range 34- 77%) fidelity during baseline with a high degree of variability. He averaged 92% (range 84–100%) during intervention and required three sessions to mastery. The data illustrate clear immediacy with a high magnitude throughout intervention. Tau-U was 1.0, a very large effect size. Max (the child participant) averaged 35% (range 18–45%) steps completed independently in baseline with a fairly stable trend. He averaged 45% (range 27–55%) during intervention with a moderate degree of variability. Tau-U was 0.56, a moderate effect size.

### Usability Survey Results

Table 3 displays detailed results from the System Usability Scale survey completed by the therapist participants after the completion of the study. The averages for items on the survey ranged from 3.25–5 for each item, which displays a relatively positive assessment of the intervention. Overall, the therapists found GAINS helpful, useful, enjoyable, easier, and more accurate than pen and paper methods. The items that scored the highest involved the ease of use of audio and visual instructions. The items scoring the lowest involved differentiating between the levels of guidance provided.

**Table 3:** Usability Survey Results

Question	Mean (Range)
The use of GAINS can enhance my job performance in helping children learn new skills	4.5 (4–5)
The use of GAINS can make me more effective in helping children learn new skills	4.5 (4–5)
The use of GAINS in helping children learn new skills enhances my productivity	4 (3–5)
Generally, I consider GAINS can be useful to me in helping children learn new skills	4.25 (4–5)
Learning to use GAINS would be easy for me	4.25 (4–5)
I find it easy to interact with GAINS	4.25 (4–5)
Interaction with GAINS is clear and easy to understand for me	4.25 (4–5)
Generally, I consider GAINS easy to use	4.25 (4–5)
If I have a choice, I intend to use GAINS in helping children learn new skills	4 (3–5)
I predict I will use GAINS in helping children learn new skills	3.5 (3–4)
If I have a choice, I plan to use GAINS in helping children learn new skills	3.75 (3–5)
I am able to integrate GAINS in helping children learn new skills	4.25 (4–5)
I can use GAINS even if there is no one to help me	3.75 (2–5)
It is easy to login into GAINS	4 (3–5)
It is easy to start instruction with the child	4.5 (4–5)
I can hear the audio assistance provided	5
It is easy to know what to do next	5
Audio assistance is easy to follow	5
Audio assistance is useful	4.5 (4–5)
The display is easy to read	5
Audio is enough. It is not necessary to read the display	4 (2–5)
It is easier than pen and paper to input data	4.75 (4–5)
Data recording is more accurate than pen and paper	4.25 (4–5)
It is useful to be provided the prompt level for a step	4.67 (4–5)
It is easy to tell what the prompt level is for a step	4.67 (4–5)
It is useful that GAINS tracks mastery of a step	4.67 (4–5)
GAINS makes it easy to track mastery of a step	5
Choosing Guidance Type (detailed, brief, etc.) is easy	3.25 (3–4)
Choosing Guidance Type (detailed, brief, etc.) is useful	3.25 (3–4)

## Discussion

The primary purpose of this efficacy study was to evaluate the use of an AI application on therapists’ implementation of evidence-based strategies to teach handwashing skills to young children with IDD. Baseline implementation fidelity performance under treatment as usual conditions was moderate for all four therapist participants, with an average of 64.5% (range 54–79%) independent and accurate implementation. Once the AI application was introduced, all four therapists demonstrated an immediate increase in performance, reaching the mastery criteria in the minimum number of sessions necessary. These results demonstrate the efficacy of AI applications, such as GAINS, in increasing treatment fidelity quickly. Although there may arguably be some learning involved while using these applications, it is not a training procedure or technology per se but rather a performance assistant designed to be used continuously. In general, the therapists found the AI system effective and easy to use. They found the data recording system to be easier to use than traditional pen and paper. The highest scoring items involved the utility and helpfulness of the audio and visual directions. One notable finding is that all therapists strongly agreed that the AI application made it easy to track mastery of a step, which could be extremely valuable when making real-time decisions on what level of prompting to use. All therapists either agreed or strongly agreed that learning to use the AI application would be easy. If therapists perceive the application as easy to use, they may be more willing to use it consistently, resulting in higher fidelity levels and improved student outcomes.

As a secondary purpose, this study evaluated the acquisition of independent handwashing by the child participants paired with each therapist. Unlike the therapists, the child participants did not demonstrate rapid acquisition. One of the participants (Mason) met the mastery criteria of 100% independent and accurate responding and two others showed slight increases in independent handwashing. There are several explanations for why we did not see the rapid acquisition one would expect to accompany such a high fidelity of implementation. First the inclusion criteria used for recruiting participants focused on lack of independence in self-help skills and did not include other skills, such as imitation, gross motor, and fine motor manipulation skills, that might be considered prerequisite to handwashing (Cooper et al., 2020). In other words, the participant’s lack of ability to manipulate the soap or move their fingers in between each other may have prevented them from acquiring these steps in the handwashing task analysis. Second, the dosage of the intervention may have been insufficient to produce significant results (St. Joseph & Machalicek, 2022; Walmsley et al., 2013). Handwashing occurs multiple times per day, though unfortunately, the AI application was only used for a few sessions per day. Thus, more often than not, the child participant was prompted to wash his or her hands without therapist or caregiver use of the AI application and perhaps using a different prompting hierarchy or chaining procedure than the one implemented by the researchers.

Finally, the teaching strategies, including a least-to-most prompting hierarchy and total task chaining procedure, were not individualized for each participant (Deochand et al., 2019; Steinbrenner et al., 2020). Again, because this research focused on the efficacy of AI applications on procedural fidelity, it may have been the case that different prompting or chaining procedures would have been more effective for the implementation of handwashing, given the child participants’ current repertoires. The current study did not systematically include reinforcement for the child’s independent responding, unlike previous studies, such as Walmsley et al. (2013). Although the therapist generally included praise for independent responding, this was not measured or reliably implemented during the current study. It is also possible that praise did not serve as a reinforcer for all of the participants. Since reinforcement is considered an evidence-based practice (Steinbrenner et al., 2020), it could be that including individualized systems of reinforcement would have enhanced skill acquisition (Deochand et al., 2019; Walmsley et al., 2013). This may have addressed a motivational component for participants that was lacking in the current study.

Although the GAINS application has the ability to provide instruction to therapists in the moment, it can only do so based on pre-programmed algorithms. It does not possess the capabilities to analyze data and alter which intervention strategies are used with participants. For example, the current study used a least-to-most prompting hierarchy that had limited success with three of the participants. It is possible, if after three or four sessions with no progress, the implementors had used a most-to-least prompting hierarchy or model prompts, the participants would have had more success (Wertalik & Kubina, 2017). In an applied setting, we would expect the treatment manager to make these kinds of changes regularly based on data (Cooper et al., 2020). The treatment manager may also have been better able to identify reinforcers or determine the reinforcing efficacy of praise for a given participant. However, there are a myriad of factors that can impede participant progress and one of the most crucial is treatment integrity, or how well staff are following the procedures as described by the treatment manager (King-Sears & Garwood, 2020; Nelen et al., 2021; O’Donnell, 2008). With the use of AI, this factor might be eliminated, and treatment can focus on other variables. Therefore, the GAINS application is not designed to replace treatment managers but rather provide them with more data and additional tools to make treatment decisions.

Currently, there is limited empirical research to support the use of AI for behavior analytic instruction for children with autism spectrum disorder, which leads many clinicians without sufficient guidance in decision making (Hopcan et al., 2022). Although more research is needed in this area, the current study provides support that AI may be effective in training therapists to use evidence-based practices with fidelity. Because AI applications, such as GAINS, provide constant access to instructions and procedures, they may serve as a technological job aid that allows therapists to maintain high levels of fidelity over time without the need for additional training. This could significantly reduce the monetary and temporal costs of ongoing monitoring and training for therapists (Neely et al., 2016; Wainer & Ingersoll, 2013). The increased rates of fidelity and associated improvements in student outcomes may reduce staff frustration and burnout rate while providing staff with an enjoyable means of conducting therapy (Fixesen et al., 2005). Future research could explore if the use of AI applications decreases the duration of sessions in comparison with other methods. A more thorough analysis of the cost of these technologies versus the cost of traditional training methods would provide additional support to management staff in making training decisions.

In terms of limitations, this study lacked the incorporation of generalization and maintenance sessions. Determining if this skill would generalize to different children and instructional programs is an important component of any behaviorally based program (Wertalik & Kubina, 2017). Furthermore, a maintenance session (probes without the AI application) and/or long-term follow-up data collection would provide evidence as to the durability of the acquired behavior. Future studies should incorporate generalization and maintenance sessions into the research design. In addition, procedural fidelity measures were not taken, as to whether or not the primary investigator followed study procedures. However, the Implementor Fidelity Checklist served as a measure of whether or not teaching procedures, such as least-to-most prompting, total task chaining, and time delay, were implemented with fidelity for the child participants.

Another limitation is the lack of child participants’ acquisition of the target skills. Future studies should determine if the participants first demonstrate the appropriate prerequisite skill repertoires for a given target program. In other words, rather than recruiting convenience samples, specific programs should be targeted based on child participants’ repertoires and prompting strategies should be individualized based on participant characteristics. The use of evidence-based practices, such as prompting and time delay, for acquisition of handwashing skills for individuals with IDD has not been well established or explored in the literature (St. Joseph & Machalicek, 2022). Previous studies (Deochand et al., 2019; Walmsley et al., 2013) have focused on increasing the effectiveness of handwashing (i.e., duration, thoroughness, etc.) in individuals who were already able to wash their hands independently. In contrast, the current study sought to establish initial handwashing skills for young children. This could be a reason for the lack of skill acquisition in some of the child participants. Future research should explore if different instructional practices are needed to initially establish handwashing skills than to refine skills that have already been acquired. Perhaps the addition of systematic manipulations of reinforcement in combination with prompting procedures would be more effective.

In summary, the evidence shows that the AI application was efficacious in increasing treatment fidelity and that the therapists evaluated its usability positively. Because AI technology remains stable and accessible over time, it is more likely that high levels of therapist fidelity will maintain over time, thus overcoming limitations of previous studies (Neely et al., 2017). AI, such as GAINS, may best be regarded as a digital performance assistant or coach that provides ongoing guidance to the user. As behavioral programs, goals, and clients change, AI can adapt and provide guidance if it is programmed to do so. Though these results should be considered preliminary, given the limited sample size, they are promising. Future research should be conducted to establish such AI as best practice for implementation/guided instruction and data collection.

## Data Availability

Data for this study is available upon request from the first author.

## Appendix A

### Implementer Fidelity Data Sheet

1. Turn on water.
  - Did the implementer use the correct prompt level? YES NO N/A
  - Did the implementer provide 3 s for an independent response? YES NO N/A
  - Did the implementer collect data correctly? YES NO N/A
  - Did the implementer proceed to the next step in the task analysis? YES NO N/A
  - Child Response: FPP PPP G IND
2. Wet hands.
  - Did the implementer use the correct prompt level? YES NO N/A
  - Did the implementer provide 3 s for an independent response? YES NO N/A
  - Did the implementer collect data correctly? YES NO N/A
  - Did the implementer proceed to the next step in the task analysis? YES NO N/A
  - Child Response: FPP PPP G IND
3. Put soap on hands.
  - Did the implementer use the correct prompt level? YES NO N/A
  - Did the implementer provide 3 s for an independent response? YES NO N/A
  - Did the implementer collect data correctly? YES NO N/A
  - Did the implementer proceed to the next step in the task analysis? YES NO N/A
  - Child Response: FPP PPP G IND
4. Scrub palms of hands.
  - Did the implementer use the correct prompt level? YES NO N/A
  - Did the implementer provide 3 s for an independent response? YES NO N/A
  - Did the implementer collect data correctly? YES NO N/A
  - Did the implementer proceed to the next step in the task analysis? YES NO N/A
  - Child Response: FPP PPP G IND
5. Scrub backs of hands.
  - Did the implementer use the correct prompt level? YES NO N/A
  - Did the implementer provide 3 s for an independent response? YES NO N/A
  - Did the implementer collect data correctly? YES NO N/A
  - Did the implementer proceed to the next step in the task analysis? YES NO N/A
  - Child Response: FPP PPP G IND
6. Scrub between fingers.
  - Did the implementer use the correct prompt level? YES NO N/A
  - Did the implementer provide 3 s for an independent response? YES NO N/A
  - Did the implementer collect data correctly? YES NO N/A
  - Did the implementer proceed to the next step in the task analysis? YES NO N/A
  - Child Response: FPP PPP G IND
7. Rinse hands.
  - Did the implementer use the correct prompt level? YES NO N/A
  - Did the implementer provide 3 s for an independent response? YES NO N/A
  - Did the implementer collect data correctly? YES NO N/A
  - Did the implementer proceed to the next step in the task analysis? YES NO N/A
  - Child Response: FPP PPP G IND
8. Turn water off.
  - Did the implementer use the correct prompt level? YES NO N/A
  - Did the implementer provide 3 s for an independent response? YES NO N/A
  - Did the implementer collect data correctly? YES NO N/A
  - Did the implementer proceed to the next step in the task analysis? YES NO N/A
  - Child Response: FPP PPP G IND
9. Get paper towel.
  - Did the implementer use the correct prompt level? YES NO N/A
  - Did the implementer provide 3 s for an independent response? YES NO N/A
  - Did the implementer collect data correctly? YES NO N/A
  - Did the implementer proceed to the next step in the task analysis? YES NO N/A
  - Child Response: FPP PPP G IND
10. Dry hands.
  - Did the implementer use the correct prompt level? YES NO N/A
  - Did the implementer provide 3 s for an independent response? YES NO N/A
  - Did the implementer collect data correctly? YES NO N/A
  - Did the implementer proceed to the next step in the task analysis? YES NO N/A
  - Child Response: FPP PPP G IND
11. Throw paper towel away.
  - Did the implementer use the correct prompt level? YES NO N/A
  - Did the implementer provide 3 s for an independent response? YES NO N/A
  - Did the implementer collect data correctly? YES NO N/A
  - Child Response: FPP PPP G IND

## Appendix B

### Hand Washing Task Analysis

1. Turn on water.
  - Full physical prompt: Take Child’s dominant hand and turn on water faucet handle.
  - Partial physical prompt: Take Child’s elbow of dominant hand and guide toward water faucet handle.
  - Gesture prompt: Point to the water faucet handle.
  - Independent: Without help, Child turns on water faucet.
2. Wet hands.
  - Full physical prompt: Take Child’s hands and place under the water.
  - Partial physical prompt: Take Child’s elbows and guide toward the water.
  - Gesture prompt: Point to the running water.
  - Independent: Without help, Child puts hands under water.
3. Put soap on hands.
  - Full physical prompt: Take Child’s dominant hand and place under soap dispenser, putting soap onto Child’s hand.
  - Partial physical prompt: Take Child’s elbow of dominant hand and guide toward the soap dispenser.
  - Gesture prompt: Point to the soap dispenser.
  - Independent: Without help, Child puts soap on hands using the dispenser.
4. Scrub palms of hands.
  - Full physical prompt: Take Child’s hands and rub the palms together.
  - Partial physical prompt: Take Child’s elbows and guide hands together.
  - Gesture prompt: Point to the palms of Child’s hands.
  - Independent: Without help, Child scrubs the palms of hands.
5. Scrub backs of hands.
  - Full physical prompt: Take Child’s hands and put one on top of the other, rubbing them together. Repeat with the opposite hand on top.
  - Partial physical prompt: Take Child’s elbows and guide one hand on top of the other. Repeat with the opposite hand on top.
  - Gesture prompt: Point to the backs of Child’s hands.
  - Independent: Without help, Child scrubs the backs of hands.
6. Scrub between fingers.
  - Full physical prompt: Take Child’s hands and place together with fingers between each other. Rub fingers together.
  - Partial physical prompt: Take Child’s elbows and guide hands together while Child scrubs between fingers.
  - Gesture prompt: Point to Child’s fingers.
  - Independent: Without help, Child scrubs between his fingers.
7. Rinse hands.
  - Full physical prompt: Take Child’s hands and place under running water.
  - Partial physical prompt: Take Child’s elbows and guide toward running water.
  - Gesture prompt: Point to the running water.
  - Independent: Without help, Child places hands under the running water.
8. Turn water off.
  - Full physical prompt: Take Child’s dominant hand and turn off water faucet handle.
  - Partial physical prompt: Take Child’s elbow of dominant hand and guide toward the water faucet handle.
  - Gesture prompt: Point to the water faucet handle.
  - Independent: Without help, Child turns off the water faucet.
9. Get paper towel.
  - Full physical prompt: Take Child’s hands and take a paper towel from the dispenser.
  - Partial physical prompt: Take Child’s elbow of dominant hand and guide toward the paper towel dispenser.
  - Gesture prompt: Point to the paper towel.
  - Independent: Without help, Child takes paper towel from dispenser.
10. Dry hands.
  - Full physical prompt: Take Child’s hands and dry with paper towel.
  - Partial physical prompt: Take Child’s elbows and guide together with the paper towel.
  - Gesture prompt: Point to Child’s hands.
  - Independent: Without help, Child dries hands with paper towel.
11. Throw paper towel away.
  - Full physical prompt: Take Child’s hands and guide to place paper towel in the trashcan.
  - Partial physical prompt: Take Child’s elbows and guide toward the trashcan.
  - Gesture prompt: Point to the trash can.
  - Independent: Without help, Child puts paper towel in trashcan.

## Appendix C

**GAINS Social Validity Survey** We are interested in your thoughts about GAINS. As a professional who has used GAINS with a consumer, we value your input in helping us improve GAINS.

## Please place an x in the cell for your response.

**Table:**

First, we have some questions about your experience with GAINS	Question Responses				
	**Strongly Disagree**	**Disagree**	**Neutral**	**Agree**	**Strongly Agree**
The use of GAINS can enhance my job performance in helping consumers learn new skills					
The use of GAINS can make me more effective in helping consumers learn new skills					
The use of GAINS in helping consumers learn new skills enhances my productivity					
Generally, I consider GAINS can be useful to me in helping consumers learn new skills					
Learning to use GAINS would be easy for me					
I find it easy to interact with GAINS					
Interaction with GAINS is clear and easy to understand for me					
Generally, I consider GAINS easy to use					
If I have a choice, I intend to use GAINS in helping consumers learn new skills					
I predict I will use GAINS in helping consumers learn new skills					
If I have a choice, I plan to use GAINS in helping consumers learn new skills					
I am able to integrate GAINS in helping consumers learn new skills					
I can use GAINS even if there is no one to help me					

**Table:**

Now, we have questions about some features of GAINS	Question Responses				
	**Strongly Disagree**	**Disagree**	**Neutral**	**Agree**	**Strongly Agree**
It is easy to login into GAINS					
It is easy to start instruction with the consumer					
I can hear the audio assistance provided					
It is easy to know what to do next					
Audio assistance is easy to follow					
Audio assistance is useful					
The display is easy to read					
Audio is enough. It is not necessary to read the display					
It is easier than pen and paper to input data					
Data recording is more accurate than pen and paper					
It is useful to be provided the prompt level for a step					
It is easy to tell what the prompt level is for a step					
It is useful that GAINS tracks mastery of a step					
GAINS makes it easy to track mastery of a step					
Choosing Guidance Type (detailed, brief, etc.) is easy					
Choosing Guidance Type (detailed, brief, etc.) is useful					

**Table:**

Now, we have questions about GAINS and Task Analysis	Question Responses				
	**Strongly Disagree**	**Disagree**	**Neutral**	**Agree**	**Strongly Agree**
GAINS improves Task Analysis instruction					
It is important for consumers to learn job skills					
Using Task Analysis to learn job skills is useful					
